# Playing games with a thrombus: a dangerous match. Paradoxical embolism from a huge central venous cathether thrombus: a case report

**DOI:** 10.1186/1476-7120-8-6

**Published:** 2010-03-16

**Authors:** Nuno Cardim, Júlia Toste, Vanessa Carvalho, Igor Nunes, Daniel Ferreira, Vanda Carmelo, Ana SN Oliveira, José Ferro, Sylvie Mariana, Adelaide Almeida, Francisco P Machado, José Roquette

**Affiliations:** 1 Cardiology Department, Hospital da Luz. Av. Lusíada, 100. 1500-650 Lisbon, Portugal; 2 Neurology Department, Hospital da Luz. Av. Lusíada, 100. 1500-650 Lisbon, Portugal

## Abstract

Thromboembolism is a major cause of death in cancer patients. The association between paraneoplastic hypercoagulability of oncological patients and long-term central venous catheters (CVC) may result in CVC associated thrombosis. Patent *Foramen Ovale *(PFO), especially when associated with atrial septal aneurysm (ASA) is a risk factor for paradoxical embolism. We report a case of paradoxical embolism with stroke in an oncological patient with a huge CVC thrombus playing "ping-pong" with an hypermobile ASA with a PFO. We review the management of hypercoagulability in oncologic patients and discuss the potential role of routine transthoracic echocardiography before the implantation of long term central venous catheters to identify predisposing conditions to paradoxical embolism and select patients for anticoagulant therapy.

## Introduction

In oncological patients hemostasis abnormalities range from asymptomatic alterations to massive thromboembolism. Its clinical importance lies on the fact that it constitutes the second leading cause of death in cancer patients [1].

This paraneoplastic hypercoagulability syndrome, although not completely understood, results secondary from a complex imbalance between coagulation and fibrinolysis, for which contributes decreased fibrinogen and platelet catabolism, reduction of antithrombotic factors such as proteins S, C and antithrombin, enhanced platelet activation and aggregation [2], increased formation of thrombin, and thrombocitosis [3] secondary to overproduction of thrombopoietin and interleukin 6 by the tumor [4]. Its severity depends on several factors, including the type and stage of tumor, the effects of adjuvant chemotherapy, the presence of central venous catheters (CVC) for its administration, surgical procedures and other adjuvant therapies [5].

Long-term central venous catheters (CVCs) are commonly used in patients with cancer. Their presence may be complicated by the occurrence of CVC-associated thrombosis, defined as a mural thrombus extending from the catheter into the lumen of a vessel and leading to partial or total catheter occlusion. According to recent reviews, the incidence of symptomatic and asymptomatic CVC-associated thrombosis in cancer patients is much higher than in non selected populations and ranged between 0-28% and between 12-66%, respectively [6].

Autopsy reports suggest that a patent Foramen Ovale (PFO) may occur in 11% to 35% of the normal population [7], with a 34.3% age-related incidence for ages from 0 to 29 years, 25.4% for ages from 30 to 79 years, and 20.2% for ages between 80 and 99 years. Thompson and Evans consecutively examined 1100 autopsies and described a "probe-PFO" (measuring 0.2 to 0.5 cm) in 29% and a "pencil-PFO" (measuring 0.6 to 1.0 cm) in 6% of the cases [7].

Paradoxical embolism was first described by Cohnheim in 1877 [7] and refers to an embolus passing from the right to the left heart through a right-to-left shunt, leading to arterial embolism. A PFO accounts for 70% of all right-to-left cardiac shunts. Other reported sources include a ventricular septal defect, Ebstein's anomaly, pulmonary arteriovenous malformations, and a patent *ductus arteriosus*. With the introduction of transesophageal echocardiography (TEE) the ability to recognize intracardiac shunting has increased, leading to the description of a new clinical, impending paradoxical embolus [7].

A large PFO and the presence of an atrial septal aneurysm (ASA) have been identified, among others, as morphological features of PFO with high risk for paradoxical embolism [8].

## Case report

Female patient, 57 years, admitted due to an acute episode of aphasia without apparent motor deficits.

She had been diagnosed for a colon adenocarcinoma (*Kras wild-type*) three years before, with liver and lung metastasis, being now on chemotherapy (cetuxime and FOLFOX regimen, which includes folinic acid, 5-fluoruracil and oxaliplatin) delivered through a permanent CVC catheter implanted in the superior vena cava.

Three months before the patient had been hospitalized due to a *Listeria monocytogenes *bacteraemia, treated with antibiotics (amoxicillin-clavulamic acid + ceftriaxone). The patient recovered and was discharged without neurological deficits.

Fifteen days before the present admission the catheter had to be replaced because of fever of unknown origin.

The physical examination showed a vigil, lucid and cooperative patient, with transcortical motor aphasia (word repetition and understanding maintained), without focal motor deficits or other changes in the neurological exam.

The cranial-encephalic magnetic resonance with diffusion study showed lesions in the left frontal area with restricted diffusion, consistent with acute ischemic cerebral vascular lesion on the left middle cerebral artery territory.

The electrocardiogram was normal but the transthoracic echocardiogram suggested an ASA with a possible PFO.

**Figure 1:**
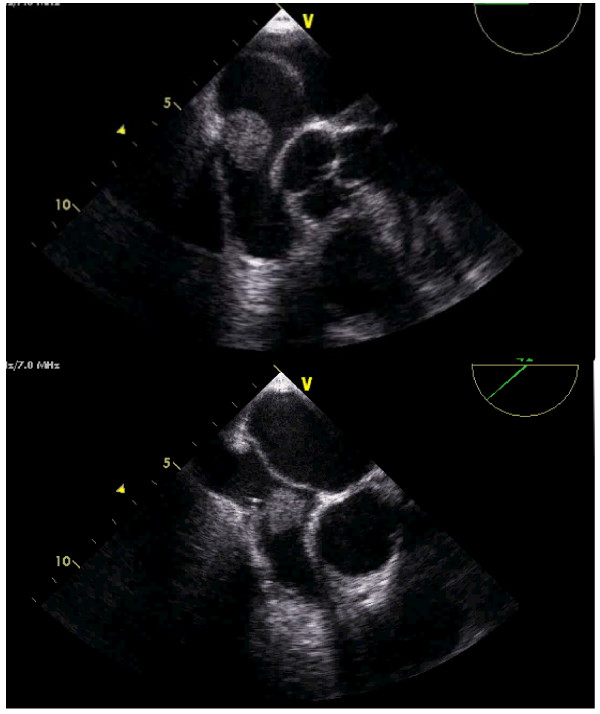
**Transesophageal echocardiography**. Huge right atrial mass. Note the hypermobile interatrial septum and the tip of the catheter in its proximity.

**Figure 2:**
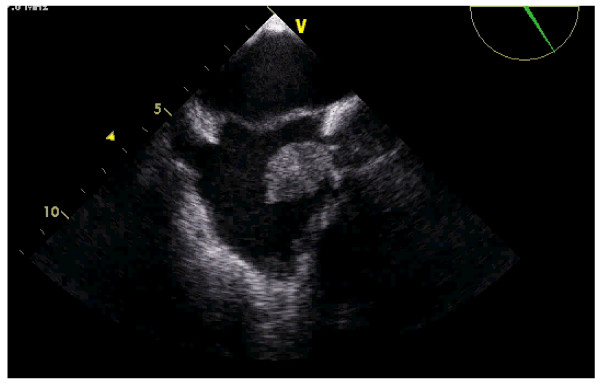
**Transesophageal echocardiography**. Bicaval modified view. The origin of the mass is in the superior vena cava, where the catheter may be seen.

**Figure 3:**
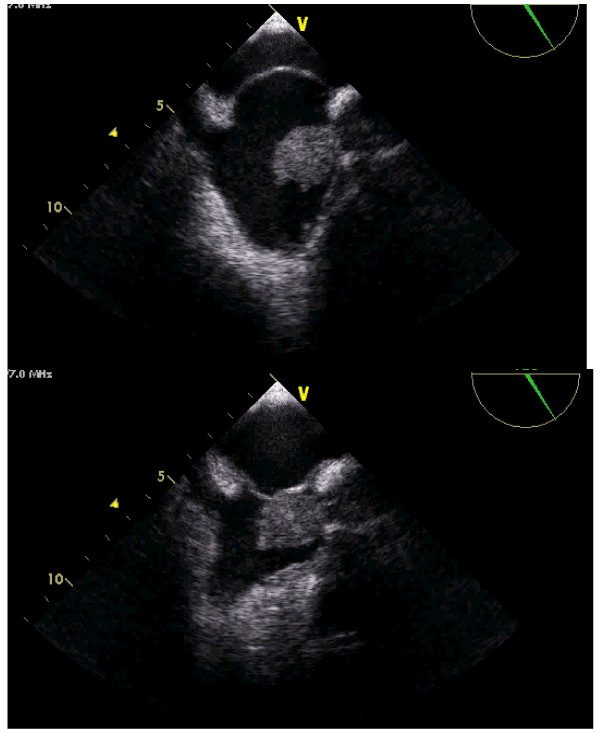
**Transesophageal echocardiography**. Playing games with a thrombus: a dangerous match. The hyperdinamic ASA beats the mass in every cardiac cycle, like a racket beating a ball in a ping-pong match.

**Figure 4:**
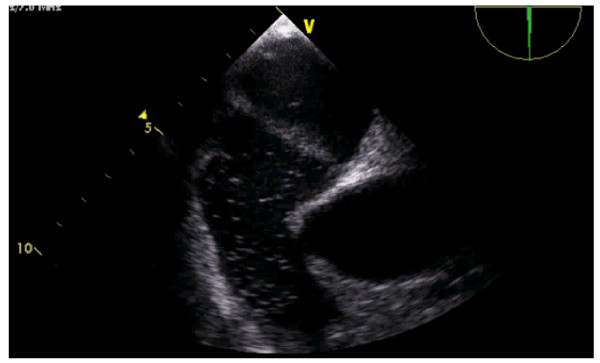
**Transesophageal echocardiography**. The injection of bolus of agitated saline in a forearm vein confirmed a small PFO, with spontaneous shunt. Note the small amount of bubbles (less than 5) in the left atrium.

The TEE showed a voluminous round mass inside the right atria (see additional file 1), coming from the superior vena cava (see additional files 2 and 3). The mass seemed to be attached to the proximal and medial part of the catheter, whose distal tip could be seen free of masses inside the right atrial cavity (Figures 1 and 2). The ASA was very mobile, hyperdinamic, beating the mass in every cardiac cycle, like a racket beating a ball in a ping-pong match (Figure 3) (see additional files 2, 3 and 4). The injection of bolus of agitated saline in a forearm vein confirmed a small PFO, with small amount of bubbles (less than 5) in the left heart without provocative Valsalva manoeuvres (figure 4).

According to the clinical data and to the results of imaging procedures described above our diagnosis was of an ischemic cerebral vascular accident (CVA) in the territory of the left middle cerebral artery by paradoxical embolism with origin in a thrombus adherent to the central venous permanent catheter through a PFO in an ASA.

**Figure 5:**
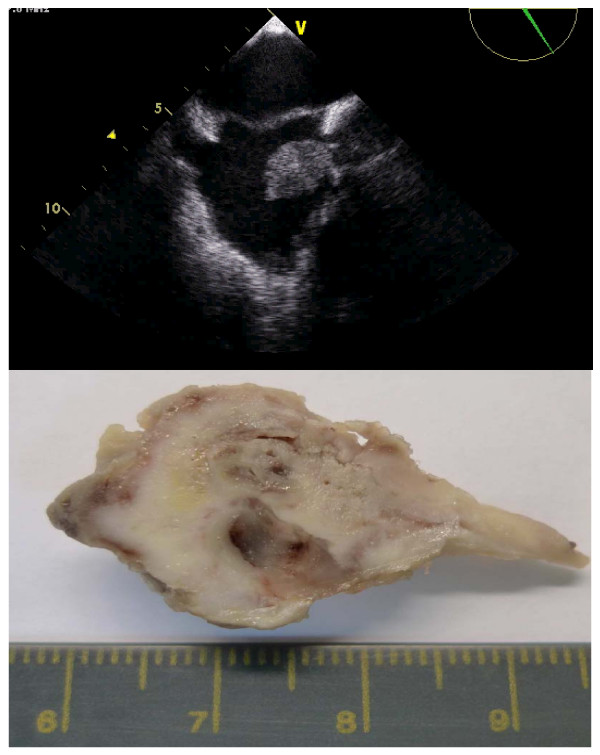
**The superior vena cava thrombus**. Note the similar shape between TEE features (top) and macroscopic data (bottom), with a long and thin border, corresponding to the insertion in the superior vena cava lumen.

The patient was empirically treated with antibiotics and anticoagulation with intravenous unfractionated heparin (target APTT 2.5× control). Full attention was given to the risk of fragmentation and multiple embolism by catheter removal and it was decided not to remove it. Since non-reduction of the thrombus with anticoagulation was seen, surgery was decided to remove the thrombus and to proceed to the closure of PFO. (Figure 5).

In the postoperative period the patient showed clinical deterioration, with diffuse abdominal pain (uncontrollable with analgesic therapy) and liver failure attributable to suprahepatic thrombosis suggesting a Budd-Chiari syndrome (documented by thoraco-abdominal CT scan that revealed significant changes involving the hepatic parenchyma, markedly heterogeneous and with bosseladura contour, associated with apparent thrombosis of the hepatic veins). The patient died about a month after surgery in multiple organ failure, after a period of coma induced by opiates for pain control.

## Discussion

This case describes the serious consequences of hypercoagulability in a patient with a solid tumor, in which the thrombotic symptoms, despite the successful removal of a giant thrombus in the right atrium, contributed inexorably to the fatal outcome. Thrombus formation is a rare complication of CVC in non-selected populations, but its incidence increases in cancer patients, as a consequence of its paraneoplastic hypercoagulability [6].

In our opinion this case report adresses several important questions:

### How to manage hypercoagulability in cancer patients with chemotherapy implanted catheters?

Is there any place for antiagregation or long term anticoagulation in cancer patients with increased prothrombotic risk with long term catheters, for which there is currently no formal recommendations? [9-14]. Schulman [11] and Smorenburg [12] suggested that, in addition to its antithrombotic mechanisms, warfarin also could have an antitumoral effect by inhibition of tumor growth and metasthasis.

Five meta-analyses [6] evaluated the efficacy and safety of Vitamin K antagonists (VKA) in the prevention of CVC-associated thrombosis, but none showed that VKA (either at a fixed low dose or with a target INR between 1.5 and 2.0) exerted a beneficial effect on the occurrence of symptomatic thromboses versus placebo or no treatment. However, in another meta-analysis, fixed low doses of VKA were more effective than placebo in preventing both asymptomatic and symptomatic CVC-associated thrombosis [relative risk = 0.37 (95% confidence interval 0.26-0.52), P < 0.001) [6]. On the other hand, and according to several randomized trials in patients with cancer, low molecular weight heparins did not show any benefit in preventing symptomatic thrombosis of the superior cava vein (though they did not increase the bleeding risk [6]).

### Which other factors increase the thromboembolic risk in oncological patients?

The chemotherapy regimen also has different prothrombotic profile. It seems that patients receiving combinations containing anthracycline, cisplatin or 5-Fluoruracil (as our patient was) have increased thrombotic risk [15] (animal experimentation microscopic studies with 5-Fluoruracil show severe endothelial damage with thrombotic formation induced by this agent [15]).

Another important issue is that our patient had previous bacteremia. Bacteremia is much more common in patients with catheter thrombosis and thrombosis is itself a risk factor for catheter infection [15].

### Was the surgical approach appropriated in this specific patient?

In the absence of guidelines, all the therapeutic decisions, specially the one of carrying on with cardiac surgery were intensively debated in multidisciplinary clinical meetings (internists, neurologists, cardiologists and cardiac surgeons). To avoid overtreatment, we followed a rational stepwise approach: our initial option was for intravenous non fractionated heparin, which was infused during 5 days without any reduction of thrombus size in a control TEE. We decided not to perform trombolysis because of the risk of thrombus fragmentation and of consequent multiple pulmonary and paradoxical systemic embolization. For the same reason we decided not to simply pull and remove the CVC, because thrombus diameter was bigger than the lumen of the superior vena cava. So, surgery was decided as the last and difficult option, taking into account the benefits (to avoid pulmonary and systemic massive embolism from a huge CVC thrombus playing ping-pong with an hyperdinamic ASA) versus the risks of the procedure.

### ASA, PFO and paradoxical embolism in oncological patients

Paradoxical embolism is a known cause of ischemic CVA in patients with PFO, embolus usually coming from venous disease of the lower limbs. However, in the reported case the origin of the thrombus was a bulky intracardiac mass diagnosed by TEE.

The clinical implications of PFO morphology and associated features are still debated [16,17]. Qualitative and quantitative and analysis by TEE is helpful in characterizing PFO-related risk of paradoxical embolism. In patients with cryptogenic CVAs large and tunnelled PFO, with "spontaneous" and severe shunt, with concomitant ASA and Chiari network increased the embolic risk [17]. So, these detailed morphological and functional data should be provided in the TEE assessment of a PFO, and not only its simple presence or absence.

## Conclusion

Thromboembolism is the second major cause of death in cancer patients and central venous catheter thrombosis is very frequent in oncological patients.

Routine transthoracic echocardiography may play an important role in the work-up of oncological patients with central venous catheters. This technique may identify predisposing conditions to paradoxical embolism and select patients for antiagregant or anticoagulant therapy. Moreover, in the presence of stroke, if an atrial septum aneurism and a patent *Foramen Ovale *is detected, a transesophageal echocardiogram must be performed for detailed morphological and functional definition of the PFO related-risk for paradoxical embolism.

In the presence of a high-risk PFO the clinical situation should be discussed in a multidisciplinary medical team (cardiologist, oncologist, neurologist) and the decision to start anticoagulation or not should be taken on an individual basis, taking into account the benefits and risks of the procedure.

## Declaration of Competing interests

The authors declare that they have no competing interests.

## Consent

A copy of the written consent is available for review by the Editor-in-Chief of this journal.

## Authors' contributions

C N carried out TEE and conceived of the paper and participated in its design and coordination and helped to draft the manuscript; T J participated in the sequence alignment and drafted the manuscript; C V participated in the sequence alignment and drafted the manuscript; F D helped to draft the manuscript; N I provided clinical medical assistance to the patient; C V provided clinical assistance to the patient; O A provided neurologic assistance; F J provided neurologic assistance; M S participated in TTE and TEE; A A, participated in TTE and TEE; M F participated in the multidisciplinary clinical meetings; R J carried out cardiac surgery.  All authors have read and approved the final manuscript.

## Supplementary Material

Additional file 1 **The thrombus**. Transesophageal echocardiography. Huge right atrial mass. The origin of the mass is not seen in this view. Click here for file

Additional file 2 **Localizing the mass**. Transesophageal echocardiography. Bicaval modified view. The origin of the mass is in the superior vena cava, where the catheter may be seen. Click here for file

Additional file 3 **Playing games with a thrombus: a dangerous match**. Transesophageal echocardiography:The hyperdinamic ASA beats the mass in every cardiac cycle, like a racket beating a ball in a ping-pong match. Click here for file

Additional file 4 **Playing games with a thrombus: a dangerous match**. Transesophageal echocardiography:The hyperdinamic ASA beats the mass in every cardiac cycle, like a racket beating a ball in a ping-pong match. Note some thrombus fragmentation. Click here for file
